# Metabolic syndrome

**DOI:** 10.1186/1824-7288-41-S2-A26

**Published:** 2015-09-30

**Authors:** Claudia Della Corte, Arianna Alterio, Valerio Nobili

**Affiliations:** 1 Hepatometabolic Department, “Bambino Gesù” Children's Hospital, Rome, Italy

## 

The epidemic spread of obesity in the last twenty years has led in pediatric setting to the appearance of diseases previously considered a prerogative of adulthood, such as metabolic syndrome (MetS).

The Metabolic Syndrome is characterized by a cluster of cardiometabolic abnormalities, including visceral obesity, dyslipidemia, hypertension and diabetes mellitus type 2, that directly increase the risk of develop cardiovascular disease and diabetes [1]. Although the pathophysiological mechanism underlying the development of MetS is still only partially understood, the most widely accepted hypothesis identify in insulin-resistance and excessive production of free fatty acids (FFAs) the key components in the development of this disease [2]. Currently, several definition of Metabolic Syndrome are available in pediatric setting, causing confusion and discrepancy in the identification of these patients. Several studies have clearly demonstrated that the prevalence of MetS in the pediatric age may widely vary using different definitions, ranging from 2.2% to 52.1% among different studies [3]. Moreover, in the last years, several other co-morbidities, besides those traditionally used to define Metabolic Syndrome, that are also linked to the disease, have been identified, making its definition even more difficult. Among these, mainly non-alcoholic fatty liver disease (NAFLD) and obstructive sleep disorders (OSAS) have been strictly linked to Metabolic Syndrome.

Lifestyle modification, based on regular physical exercise and a balanced diet appropriate for age, is the mainstay of therapeutic approach in children and adolescent with obesity and risk factors for MetS [4]. Behavioral intervention is often difficult to achieve and maintain and most pediatric patients require pharmacologic therapy early in their disease course.

Given the relatively recent occurrence of MS in childhood, long-term follow-up studies are not available yet. However, it is reasonable to think that the metabolic derangement observed in obese children will have dramatic repercussions on their health earlier than that observed in adults, with a consequent worsening of the prognosis in terms of morbidity and mortality when they are still youth.
